# Pseudotumor from ceramic-on-ceramic total hip arthroplasty

**DOI:** 10.1016/j.ijscr.2024.109374

**Published:** 2024-02-13

**Authors:** Corrado Ciatti, Pietro Maniscalco, Silvia Bosio, Calogero Puma Pagliarello, Giuseppe Bianchi, Fabrizio Quattrini

**Affiliations:** a U.O.C. Orthopedics and Traumatology, Guglielmo Da Saliceto Hospital, AUSL Piacenza, Italy; b University of Parma; c Pathology Unit, Onco-Hematologic Department, Guglielmo Da Saliceto Hospital, AUSL Piacenza, Piacenza, Italy; d Clinica Ortopedica III, IRCCS Istituto Ortopedico Rizzoli, Bologna, Italy

**Keywords:** Pseudotumor, Metallosis, Ceramic, Hip arthroplasty

## Abstract

**Introduction and importance:**

Total hip arthroplasty is one of the most performed surgical interventions in the world. Adverse local tissue reactions and pseudotumors are infrequent but dangerous eventualities, which are often related with metal-on-metal or metal-on-polyethylene implants. This study wants to highlight how adverse local tissue reactions and pseudotumors must be taken into consideration during the diagnostic process.

**Case presentation:**

We report the case of a patient with ceramic-on-ceramic modular total hip arthroplasty with titanium neck. 12 years after surgery, he complained of pain and swelling on the hip. Diagnostic tests revealed the presence of a bulky pseudotumor. During the revision surgery biopsy samples were taken and microscopical analysis revealed the presence of fibrous tissue, fibrin hemorrhagic collections, histiocytes and chronic inflammation due to foreign body, with dark refractive material of an exogenous nature.

**Clinical discussion:**

The possible formation of pseudotumor and metallosis reactions in hip prostheses with metal-on-metal coupling or in couplings with polyethylene is known. Many cases of pseudotumor are reported after revision of prostheses due to the breakage of ceramic components, but we did not observe any damage or corrosion of the prosthetic elements; on the other hand, we noticed an excessive retroversion of the femoral neck. It may be possible that an accurate microscopic analysis could clarify the failure of this implant.

**Conclusion:**

To date ceramic-ceramic coupling remains the gold standard in terms of resistance and durability for hip arthroplasty but there is still a gap of knowledge in the field of tribology and individual immune response mechanisms.

## Highlights

• ALTR and pseudotumors are infrequent complications in CoC THAs, but still possible.
• Many cases of pseudotumor are reported due to breakage of ceramic components
• CoC remains the gold standard in terms of resistance and durability

## 1 Introduction

Total hip arthroplasty, nowadays, is one of the most performed surgical interventions in the world [1,2]. Among the possible complications, metallosis is an infrequent but still dangerous and feared eventuality [3]. This issue is estimated to account for 5.3 % of THA complications [4]. In the literature, various cases of metallosis have been reported in relation to metal-on-metal (MoM) and metal-on-polyethylene (MoP) implants, often associated to the appearance of adverse local tissue reactions (ALTR) and the growth of pseudotumors [[5], [6], [7]]. It was estimated that the prevalence of pseudotumors and ALTR in MoM hip arthroplasties is 36 %–61 %. On the other hand, with a much lower frequency, this occurrence can instead be related with ceramic-on-ceramic (CoC) prostheses, even if with very lower rates [8]. In fact, only case reports can be found in the literature.

This study highlighted how adverse local tissue reactions and pseudotumors are infrequent complications in CoC prostheses, but still possible and to be taken into consideration when patients show certain signs and symptoms.

This case report was helded in line with the SCARE criteria [9].

## 2 Case report

We discuss the case of L.P.R., a 80 years old man known to us for bilateral hip replacement. In fact, he had received two surgeries for hip osteoarthritis, in 2010 the left side and in 2012 the right one. Both the left and right hip arthroplasties were composed of a titanium modular stem with titanium neck, a ceramic head and a titanium acetabular cup with ceramic insert. The two postoperative courses elapsed regularly and without complications, from both a clinical and radiographic point of view, with the exception of the presence of some calcifications around the left implant manifested after a few months from the first surgery.

12 years after left hip replacement, the patient begins to have swelling and left thigh pain, which started gradually during the previous two weeks, in the absence of trauma and without functional limitation. The patient was suffering from hypertension, paroxysmal atrial fibrillations and dyslipidemia; he was taking Ramipril 2.5 mg/die, Bisoprolol 5 mg/day, Flecainide 100 mg/day, Simvastatin 20 mg/day and Rivaroxaban 20 mg/day (which was substituted with Enoxaparin 4000 UI/day few days before surgery, according to cardiological advise). The patient was vaccinated with mRNA-based Covid-19 vaccine (3 doses).

**Fig. 1:**
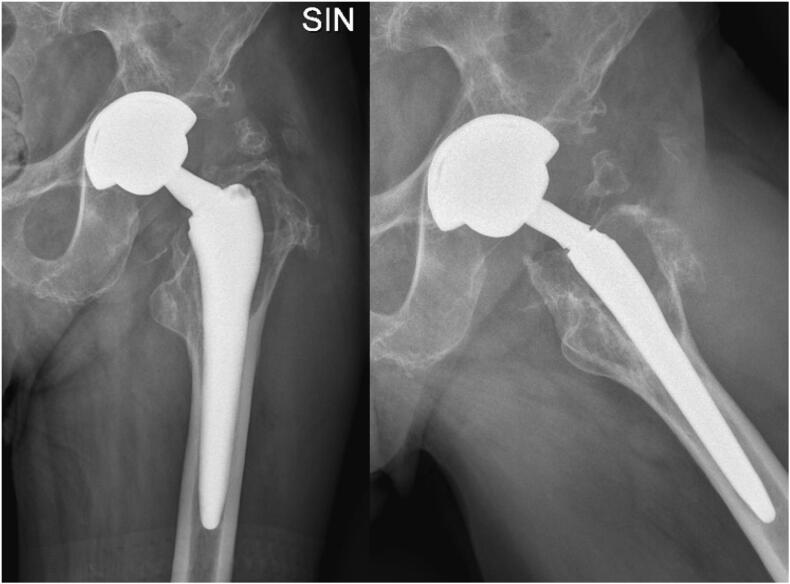
Preoperative X-rays (anteroposterior and lateral view) - bone resorption around the left femoral stem.

**Fig. 2:**
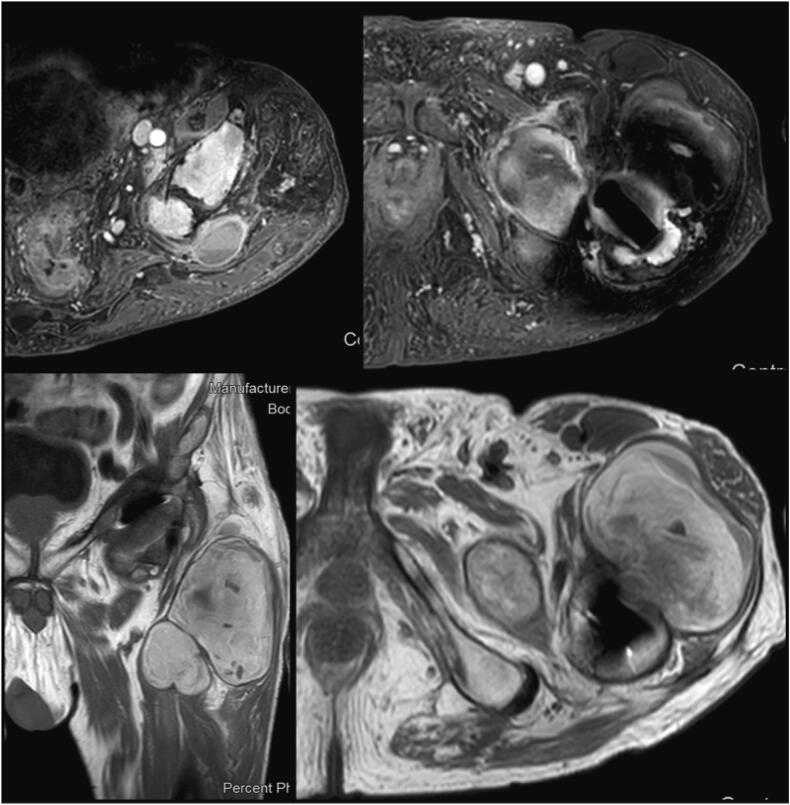
Preoperative MRI, Adverse Local Tissue Reaction and pseudotumor - bone resorption around the left femoral stem, left ilium and the acetabulum; protein/hemorrhagic subsolid collection of 7 cm in diameter inside the proximal part of the quadriceps muscle, which involved also the iliopsoas bursa and extended to the pelvis.

Haematological examinations did not report active or chronic infections, inflammatory and tumor markers were off (WBC 5000/mm^3^, CRP 0.54 mg/dL, ESR 22 mm/h, CEA 2.35 ng/mL, CA 19-9 9 UI/mL, CA 15-3 22 UI/mL, CA 125 29 UL/ml). The patient underwent to an ultrasound examination that showed a voluminous hematoma being organized. X-rays evaluation of the left hip displayed bone resorption around the left femoral stem (Gruen zone 1) (Fig. 1). Therefore, it was decided to refer the patient both pelvis CT scan and MRI, which confirmed the pathological findings in the Gruen zone 1, reporting also bone resorption at the wing of left ilium and the acetabulum; anteriorly, inside the proximal part of the quadriceps muscle, there was a coarse, protein/hemorrhagic, subsolid collection of 7 cm in diameter, which involved also the iliopsoas bursa and extended to the pelvis (Fig. 2). White and red blood cell count, serum C-reactive protein and erythrocyte sedimentation rate were normal, without clinical signs of sepsis; Cobalt and chromium levels were found to be normal, respectively at 0.3 and 0.35 ppb.

Consequently, the patient was hospitalized and an ultrasound-guided fine needle aspiration of the periprosthetic collection was performed. The results ruled out malignant tumor, instead raising the suspicion of periprosthetic infection or metallosis, that not even the scintigraphy resolved. In addition,

Assuming it was an ALTR with possible formation of a pseudotumor, we planned for an implant substitution with pseudotumor removal [10]. However, during the hospitalization the patient contracted Covid-19. In accordance with strict internal protocols on the subject of coronavirus disease, due to the non-emergency/urgency nature of the case, it was necessary to wait for the nasopharyngeal swab to be negativized before proceeding with surgery. The operation was performed 4 days after hospitalization [11,12].

**Fig. 3:**
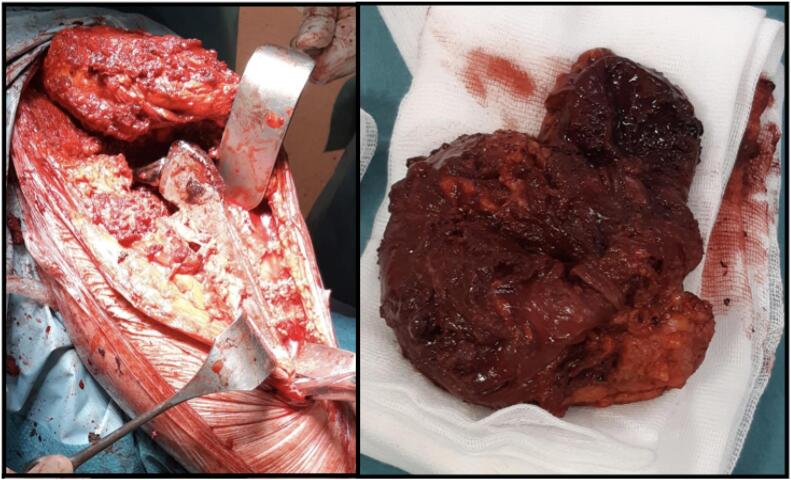
Intra-operative images: through the Enneking approach and the detachment of the gluteal muscles from the ileum, the pseudotumor was removed.

**Fig. 4:**
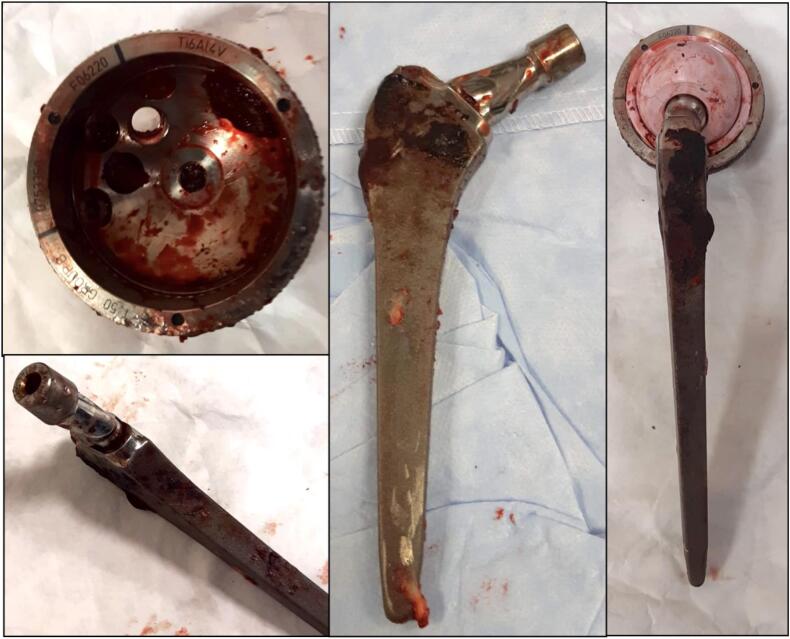
Explanted components, the femoral stem through Wagner's trans-femoral access, the acetabular cup with the help of gauges.

**Fig. 5:**
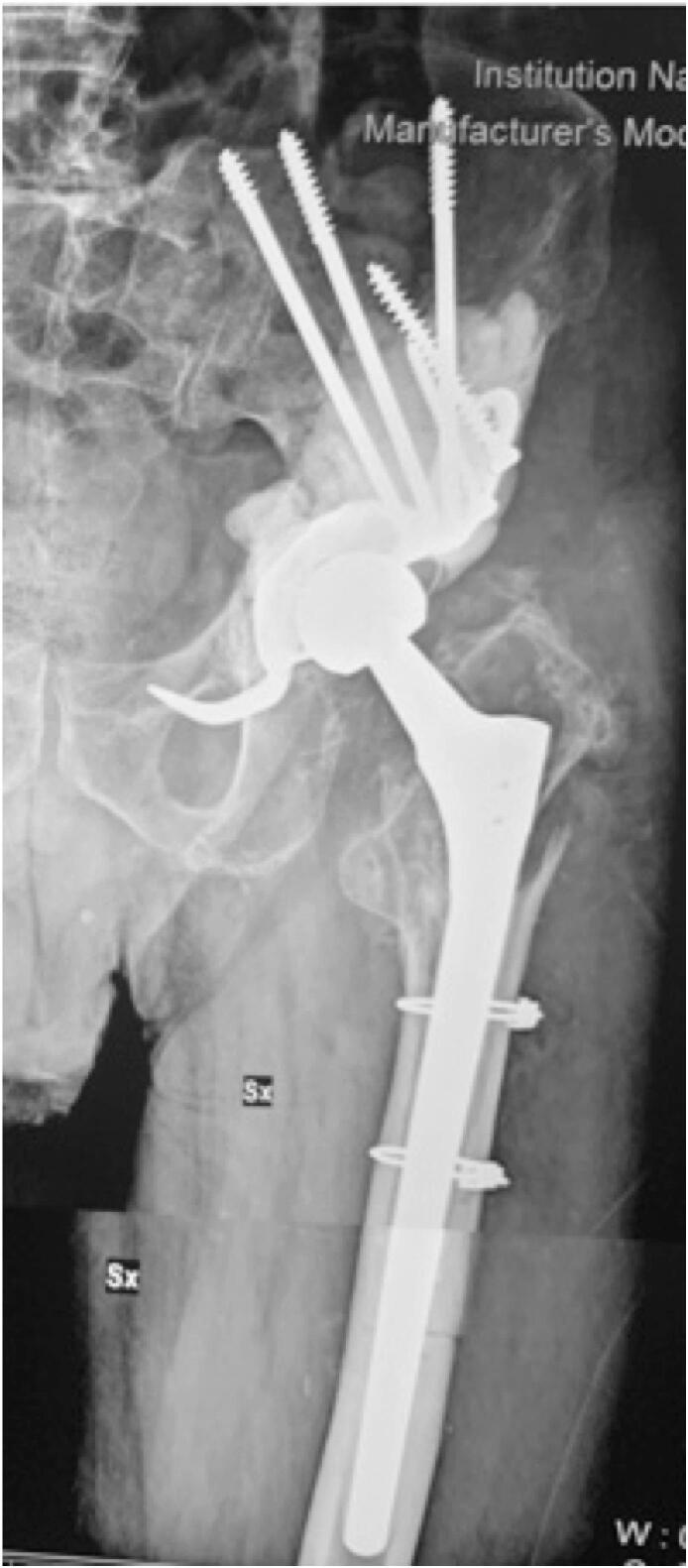
Post-operative result; Burch Schneider cage with screws and filler cement, Muller II cemented cup (Smith & Nephew), 225 mm monolithic Wagner SL Revision Hip Stem (Zimmer Biomet) with a 32 mm femoral head, two cerclages.

During surgery, the patient was placed in lateral position. Through the Enneking approach and the detachment of the gluteal muscles from the ileum, we accessed the articular cavity. Raising the lateral vastus, the voluminous polylobulated formation could be appreciated together with the erosion of the anterior proximal metaphysis that it had caused. The anterior component of the pseudotumor was excised, also taking some samples, exposing the prosthetic implant (Fig. 3). The components appeared osseointegrated, so the femoral stem was explanted through Wagner's trans-femoral access and the acetabular cup with the help of gauges (Fig. 4). A broad osteolysis was highlighted in the supracetabular portion, which extended up to ileum and ischium. To reconstruct the acetabular side, a Burch Schneider cage with four screws and filler cement was used, in addition to a Muller II cemented cup (Smith & Nephew, London, UK). At the femoral side, a 225 mm monolithic Wagner SL Revision Hip Stem (Zimmer Biomet, Warsaw, IN, USA) was employed, with a 32 mm femoral head and a medium neck, closing the femoral canal with two cerclages (Fig. 5).

Microscopical analysis of the intra-operative samples revealed the presence of fibrous tissue, fibrin hemorrhagic collections, histiocytes and chronic inflammation due to foreign body, with dark refractive material of an exogenous nature.

The day after he was transfused with 2 units of red blood cells due to anemia (8.2 g/dL). During the first month the load was not allowed on the left side and monopodalic walking with 2 crutches was allowed, in addition to active and passive kinesis therapy of the left limb. 6 weeks after surgery, the patient was walking with 2 crutches and a partial load; no complications was reported; he did not present pain or swelling on his left hip.

**Fig. 6:**
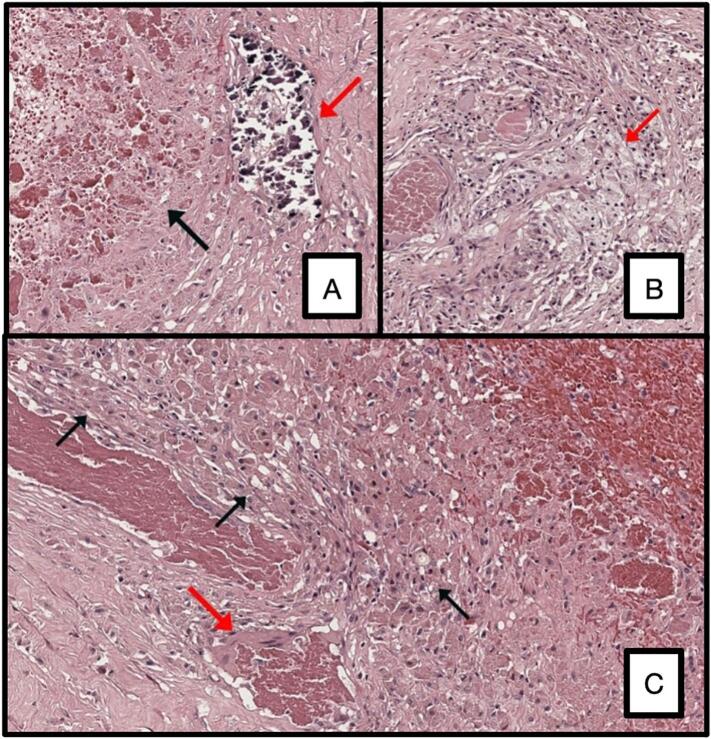
Microscopic analysis. A. Red arrow, exogenous material refracting at light microscopy. Black arrow, fibrin-hematous material. H&E, 20× magnification. B. Red arrow, granulomatous reaction with foamy hystiocytes. Pigmented macrophages can be seen scattered around this formation. H&E, 20× magnification. C. Black arrows, hystiocytes wall surrounding fibrin-hematous material (top right). Red arrow, a giant multi-nucleated cell. H&E, 20× magnification. (For interpretation of the references to colour in this figure legend, the reader is referred to the web version of this article.)

Microscopic analysis found out that the specimen consisted of fibrous, adipose, and striated muscle tissue with massive fibrin-hematous collections surrounded by a histiocyte wall; few granulomatous aggregates of foamy macrophages, scattered histiocytes containing hematin pigment, plurinucleated giant cells, and deposits of blackish material refracting on light microscopy, interpreted as exogenous, were observed nearby (Fig. 6).

## 3 Discussion

The possible formation of pseudotumor and metallosis reactions in hip prostheses with metal-on-metal coupling or in couplings with polyethylene is well known and high incidences are reported in some cases [[5], [6], [7]].

To date the use of ceramic-on-ceramic coupling is currently considered the gold standard for implant survival thanks also to the absence of macroscopic debris products or ion releases that could lead to local and systemic reactions [13,14]. However, even this coupling may be subject to complications, including the possible fracture of the head the liner (1,3/100.000, that is greatly decreased over the years) and the so-called squeaking, that is, the generation of audible noise, which in most cases is a transient condition [15].

The increase in the number of interfaces, as in the case of using modular components, could theoretically boost the possibility of debris formation and ion release, raising the risk of implant failure [[16], [17], [18]]. However, in our previous study in which we checked over 900 patients with modular prosthetic implants with an average follow-up of approximately 10 years, none of the patients with ceramic-ceramic bearing was found to have a pseudotumor [19]. We hypothesized that the exclusive use of titanium modular necks prevented these phenomena. In fact, given the different characteristics of the materials, the coupling of titanium stems and cobalt chrome necks gave disastrous results, with implant failures, metal ion generations, ALTRs and bulky pseudotumor formations [16,20,21].

In literature, many cases of pseudotumor are reported after revision of prostheses due to breakage of ceramic components [14,[22], [23], [24], [25]]; only one case of ALTR in a patient with first implant ceramic-on-ceramic prosthesis [8]. We didn't report any evident breakages of the implant components or scratches or wear damage that could justify a release of metal ions and debris; prosthetic stem and femoral neck were completely fused and inseparable without signs of local wear or damage.

About the pseudotumor pathopisiology in CoC THAs, most of the cases presented in the literature are caused by the breakage of the ceramic head or liner, which leads to progressive wear. Moreover, also a significant malposition of a ceramic component may cause an alteration of loading and an excessive wear [8].

During surgery we noticed an excessive retroversion of the femoral neck, but we cannot be sure that this has affected the prosthetic failure, as there were no signs of impingement and/or wear on the prosthetic components.

Therefore, it was not possible to identify with certainty the specific cause that led to the formation of the local adverse reaction and the consequent symptom pattern. On the other hand, it may be possible that an accurate microscopic analysis could clarify the failure of this implant.

To date ceramic-ceramic coupling remains the gold standard in terms of resistance and durability for hip arthroplasty but there is still a gap of knowledge in the field of tribology and individual immune response mechanisms.

## Consent

Written informed consent was obtained from the patient for publication of this case report and accompanying images. A copy of the written consent is available for review by the Editor-in-Chief of this journal on request.

## Ethical approval

The study is exempt from ethnical approval since it is retrospective case report, whose data is totally anonymized.

## Funding

The authors declare that they did not receive any funding for this study.

## Author contribution

Conceptualization, F.Q., C.P.P. and C.C.; methodology, F.Q. and C.C.; validation, P.M.; investigation, F.Q., S.B. and C.C.; writing—original draft preparation, F.Q., C.P.P. and C.C.; writing—review and editing, F.Q., S.B. and C.C.; visualization, P.M. and G.B.; supervision, P.M and G.B.

## Guarantor

Ciatti Corrado, Pietro Maniscalco, Fabrizio Quattrini.

## Conflict of interest statement

All other authors declare that they have no existing conflicts of interest.
